# Angiogenic Factors Stimulate Growth of Adult Neural Stem Cells

**DOI:** 10.1371/journal.pone.0009414

**Published:** 2010-02-26

**Authors:** Andreas Androutsellis-Theotokis, Maria A. Rueger, Deric M. Park, Justin D. Boyd, Raji Padmanabhan, Loraine Campanati, Craig V. Stewart, Yann LeFranc, Dietmar Plenz, Stuart Walbridge, Russell R. Lonser, Ronald D. G. McKay

**Affiliations:** 1 Laboratory of Molecular Biology, National Cancer Institute, National Institutes of Health, Bethesda, Maryland, United States of America; 2 Surgical Neurology Branch, National Institute of Neurological Disorders and Stroke, National Institutes of Health, Bethesda, Maryland, United States of America; 3 Laboratory of Systems Neuroscience, National Institute of Mental Health, National Institutes of Health, Bethesda, Maryland, United States of America; 4 Laboratory of Cell Biology, National Cancer Institute, National Institutes of Health, Bethesda, Maryland, United States of America; City of Hope Medical Center and Beckman Research Institute, United States of America

## Abstract

**Background:**

The ability to grow a uniform cell type from the adult central nervous system (CNS) is valuable for developing cell therapies and new strategies for drug discovery. The adult mammalian brain is a source of neural stem cells (NSC) found in both neurogenic and non-neurogenic zones but difficulties in culturing these hinders their use as research tools [1], [2], [3], [4], [5], [6].

**Methodology/Principal Findings:**

Here we show that NSCs can be efficiently grown in adherent cell cultures when angiogenic signals are included in the medium. These signals include both anti-angiogenic factors (the soluble form of the Notch receptor ligand, Dll4) and pro-angiogenic factors (the Tie-2 receptor ligand, Angiopoietin 2). These treatments support the self renewal state of cultured NSCs and expression of the transcription factor Hes3, which also identifies the cancer stem cell population in human tumors. In an organotypic slice model, angiogenic factors maintain vascular structure and increase the density of dopamine neuron processes.

**Conclusions/Significance:**

We demonstrate new properties of adult NSCs and a method to generate efficient adult NSC cultures from various central nervous system areas. These findings will help establish cellular models relevant to cancer and regeneration.

## Introduction

The identification of precursor cells in the adult nervous system raises the possibility that clinical benefit may be achieved via their activation. Scientific interest has mostly focused on neural precursors in the sub-ventricular zone (SVZ) and the dentate gyrus (DG) of the hippocampus that continually give rise to new neurons [3], [7], [8], [9], [10], [11], [12], [13]. In the SVZ and DG, the endogenous neural stem cell and neural precursor population can be induced to expand by injury [14], [15], [16], [17], [18], [19] and by pharmacological manipulations that include treatments with basic Fibroblast Growth Factor (EGF), Epidermal Growth factor (EGF), Platelet-Derived Growth Factor (PDGF), insulin, and perturbation of ephrin signaling [10], [11], [20], [21], [22], [23], [24], [25]. However, the low yield of current adult neural stem cell culture systems hinders the efforts to identify novel treatments and elucidate the key signal transduction steps that regulate neural stem cell numbers in vitro and in vivo. Our previous work on Notch, insulin, and angiopoietin signaling in neural stem cells provided us with a signal transduction logic that we used here to improve neural stem cell culture yields.

The Notch receptor and its ligands are known to regulate the development and maintenance of neuroepithelial precursors and brain vasculature [26], [27], [28], [29], [30]. We have previously reported that a novel signal transduction pathway downstream of the Notch receptor expands neural stem cell populations both in vitro and in vivo [31]. This pathway links the Notch receptor to phosphorylation of STAT3 on the serine residue and a subsequent rapid increase in the transcription factor Hes3 that causes a long lasting elevation of sonic hedgehog (Shh). Delivery of the Notch ligand Deltalike-4 (Dll4) to the adult CNS increases the number of precursor cells in the SVZ and confers behavioral benefits in a model of ischemic stroke [31].

The angiopoietins (Ang1 & Ang2) are important regulators of endothelial and hematopoietic stem cells [32], [33], [34], [35]. We have previously shown that precursors in the SVZ and throughout the brain express the angiopoietin receptor Tie2. Single intraventricular injections of Dll4 or Ang2 increase the number of Hes3-positive immature cells that are widely distributed outside the neurogenic regions in the adult rat and primate CNS [6]. Similar to Dll4, Ang2 also induces phosphorylation of STAT3 on the serine residue, in fetal neural stem cell cultures. In the rat, treatments that increase the numbers of Hes3 cells (Dll4, Ang2, insulin) also rescue midbrain dopamine neurons from a lethal toxin, 6-hydroxy-dopamine (6OHDA). Animals with increased numbers of Hes3 cells also show a sustained behavioral improvement that is dependent on dopamine neuron function [6], [25]. Here we used various treatments to establish efficient cultures of neural stem cells from different areas of the adult rodent and monkey central nervous systems.

## Results and Discussion

**Figure 1:**
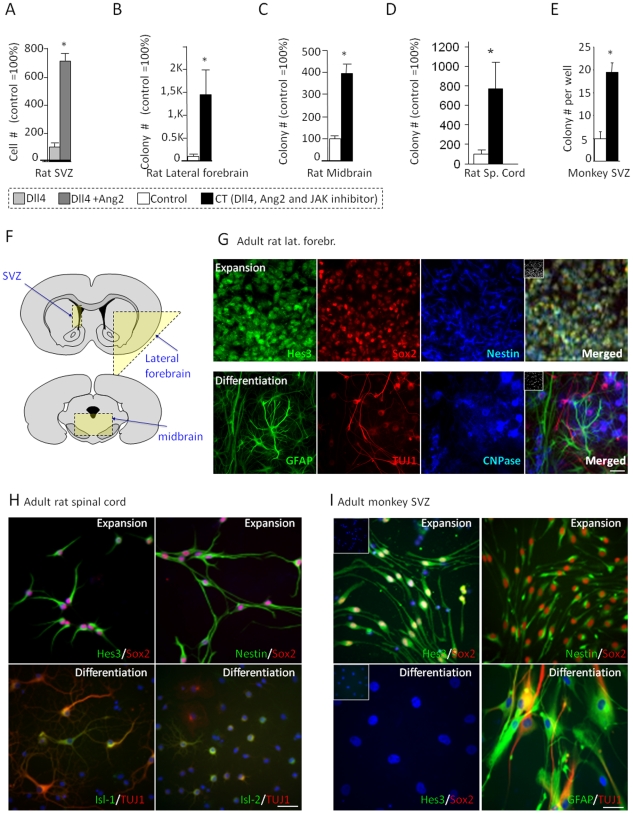
Efficient culture of adult NSCs by angiogenic factors. (A–E) Treatments increase rat and monkey adult neural precursor expansion in vitro (Rat, 5-d expansion; Monkey, 10-d). (F) Diagram of the areas dissected for the rat cultures. (G) Precursor and differentiation marker expression in the expansion and differentiation (by 2-weeks mitogen withdrawal) stages from rat lateral forebrain. (H) Precursor and differentiation marker expression in the expansion and differentiation (by a 10-day mitogen withdrawal) stages from rat spinal cord. (I) Precursor and differentiation marker expression in the expansion and differentiation (by 2-weeks mitogen withdrawal) stages from the adult monkey SVZ. [Size bars: 20 µm].

The observation that angiogenic factors expand the endogenous neural stem cell and precursor population suggests that angiogenic growth factors may promote the efficient growth of NSCs from the adult brain in adherent cell culture [25], [36]. We used an established method to grow NSCs as adherent monolayers [37]. The culture medium contains insulin and the mitogen basic Fibroblast Growth Factor (FGF2). Here we studied the effects of adding angiogenic factors to the culture medium on NSC numbers from different areas of the CNS. When cells from the adult rat SVZ were placed in culture, Dll4 increases the number of NSCs [31]. When Ang2 was included in the medium in addition to Dll4, a further 7-fold increase in the numbers of proliferating precursors was obtained (Figure 1A). [Numbers represent cell number as a percentage of control condition cultures].

Whether NSCs exist in non-neurogenic zones in the adult brain at significant numbers has been contested. The improved efficiency of our adult cultures prompted us to ask if cultures could be established from these areas. A combination treatment that includes Dll4, Ang2, and a Jak kinase inhibitor, previously shown to increase NSC expansion in vivo [31] (Combination Treatment, “CT”) supports cell growth from the lateral regions of the forebrain and the ventral midbrain (Figure 1B, C, F). Total cell number and the number of colonies were both increased by the combination of factors (data not shown). CT also promoted expansion of NSCs from the adult rat spinal cord (Figure 1D) and the adult monkey SVZ (Figure 1E). All proliferating cells expressed the NSC markers nestin [38], Sox2 [39], and Hes3 [6] (Figure 1G–H). When grown at low (clonal) density and switched into conditions that support differentiation, the precursor markers were lost and the cells acquired morphologies and antigen expression patterns found in neurons, astrocytes, and oligodendrocytes (Figure 1G–H; Table 1). [Numbers represent colony number as a percentage of control condition cultures. Absolute values of Hes3+ cells in different areas of the adult brain are presented in Table 2]. Although the need for micro-dissection to establish adult NSC cultures from the adult brain makes a quantitative comparison of the yield from various areas difficult, the expansion of colonies from all areas followed a similar time course; confluence within individual colonies was reached within approximately one week.

**Table 1:** Differentiation potential of adult SVZ and lateral forebrain precursors.

	%Neurons (TUJ1)	%Glia (GFAP)	%Oligodendrocytes (CNPase)
**SVZ**	43±10	51±10	9±2
**Lateral forebrain**	37±7	32±4	30±3

Ratios of neurons (TUJ1+), astrocytes (GFAP+), and oligodendrocytes (CNPase+) following Notch activation (7-d Notch + FGF2, 10-d withdrawal).

**Table 2:** Prevalence of Hes3+ cells in adult rat central nervous system areas.

Area	Hes3+ cell#/field	SD
SVZ	9.4	4.4
Striatum	0.5	0.4
Substantia Nigra	0.9	0.8
Hippocampus	0.7	0.7
Cerebral cortex	0.4	0.2
Lateral forebrain	1.3	1.2
Aqueduct	3.7	1.3
Spinal Cord	0.4	0.3

Adult rats were perfused and 16 micrometer – thick sections were prepared from the brain and spinal cord. The sections were immunolabeled for Hes3 and the Hes3+ cell numbers were counted using a fluorescent microscope with a x40 objective. The field of view is 0.05 micrometers squared.

NSCs from the adult rodent and monkey brain express Hes3; differentiation causes a rapid loss of Hes3 expression [6]. In vitro, established fetal NSCs also express receptors for Dll4 and the angiopoietins [6], [31]. These cells also express the angiopoietins themselves. Under conditions that support self-renewal (in the presence of FGF and FGF + Dll4, for 2 days), fetal NSCs express both Ang1 and Ang2. In contrast, when NSCs are induced to differentiate by the addition of Ciliary Neurotrophic Factor (CNTF), expression of Ang2 is rapidly lost (Figure S1). In the brain, Hes3+ cells are in close proximity to blood vessels [6], which are a source of both the angiopoietins and Dll4. In vitro, Ang2 induces STAT3-Ser phosphorylation and we have shown that this event leads to Hes3 transcription and subsequent sonic hedgehog expression [31]. In interstitial mesenchymal cells, sonic hedgehog induces the expression of the angiopoietins [40]. An intriguing possibility is that a positive feedback loop involving Ang2, STAT3-Ser, Hes3 and sonic hedgehog contributes to the self renewal, survival, and expansion of neural stem cells.

**Figure 2:**
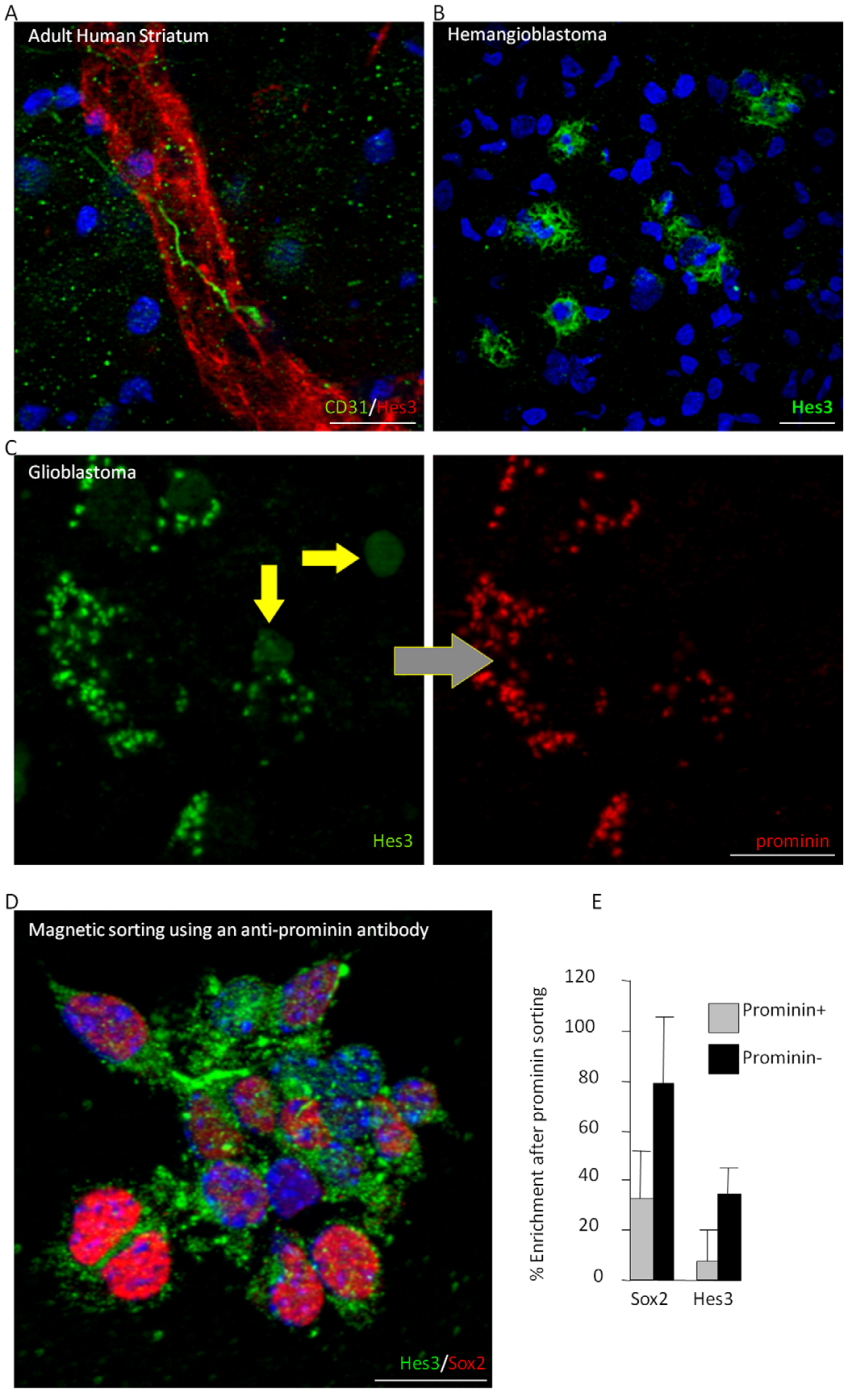
Hes3 is a marker of normal and cancer human stem cells. (A–C) Hes3^+^ cell in the striatum of non-cancerous adult human brain tissue (blood vessels identified by RECA-1 expression), human hemangioblastoma (HBM) biopsy (HBM Hes3^+^ megakaryocytes shown), human glioblastoma multiforme (GBM) biopsy (Hes3 co-expressed with prominin). (D) Fetal cortical cells sorted for prominin express Sox2 and Hes3. (E) Enrichment for Sox2+ and Hes3+ cells by magnetic sorting using an anti-prominin antibody. (Size bar, 50 µm).

Here, in addition to previously defined self-renewal markers, we used Hes3 expression to assess fast changes in cell state and to determine if Hes3 also recognizes cancer stem cells arising in the CNS. Antibodies against the tetraspan protein prominin have also been widely used to isolate somatic and cancer stem cells [41], [42]. Precursor cells in human tumor and non-cancerous tissue co-express Hes3 (Figure 2A–C). Fetal mouse NSCs prospectively isolated by prominin expression are six fold enriched for Hes3 expression (6.42±1.9, n = 4; Figure 1D, E). This report shows that a multipotent precursor cell that is widely distributed in the adult brain shares common marker expression with stem cells found in CNS cancers.

Dll4 and Ang2 both support the growth of NSCs but have opposite effects on angiogenesis [6]. These observations show the importance of understanding the actions of angiogenic factors on NSCs in the context of the multiple cell types found in adult tissue, including the cells of the vascular system. Explants of the Substantia Nigra (S. Nigra), striatum and cerebral cortex are grown in co-culture; cortical glutamatergic and midbrain dopamine neurons extend axons into the striatum where they generate synapses with the appropriate physiological properties seen in vivo [43]. The effects of angiogenic growth factors in this organotypic cell culture system were defined.

**Figure 3:**
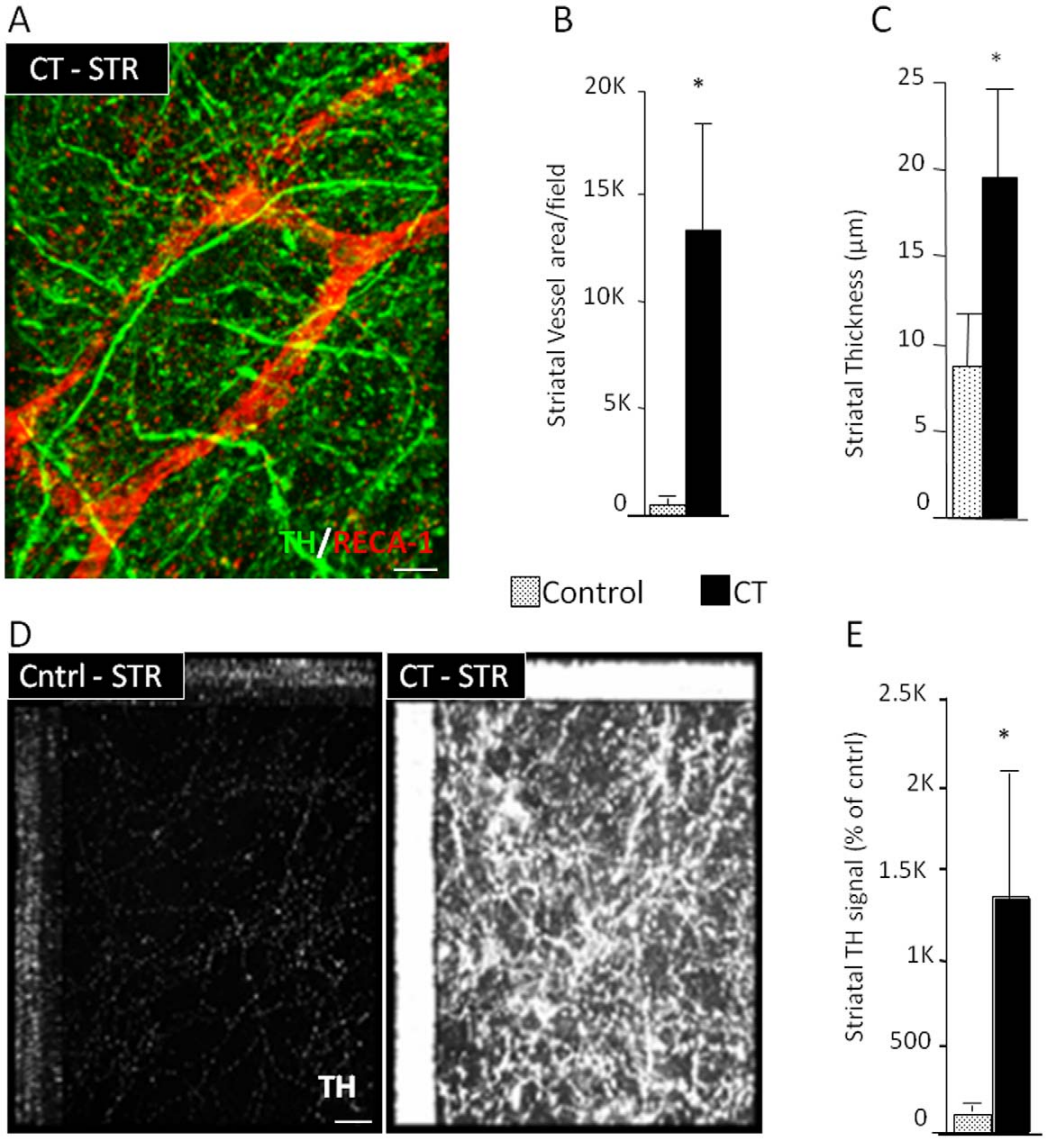
Increased vascular coverage and neuronal projections by angiogenic factors. (A, B) CT treatment of organotypic slice cultures (every 4 days for 2 weeks) retains the vasculature (confocal projection for the pan-endothelial marker RECA-1 and TH), (C) increases the thickness of the striatal portion of the slice, (D,E) promotes the sprouting of TH^+^ fibers from the S. Nigra section to the striatal section (2-weeks after control (BSA) and CT treatment). [Size bars: 20 µm].

Immunohistochemistry with an antibody against a pan-endothelial marker (RECA-1) and quantitation by pattern recognition software (Zeiss Axiovision) confirmed a marked increase in the density of blood vessels in CT - treated striatal explants (Figure 3A, B). In contrast, there was no effect on the numbers of blood vessels in the treated S. Nigra. When the CT factors were present, the striatal tissue was thicker (Control: 8.7 µm±3.01; CT: 19.4 µm±5.3; N = 9) and there was a 10-fold increase in the density of TH^+^ processes (Figure 3C–E). In the S. Nigra, there was no change in the thickness of the slice nor was there any change in the number of TH^+^ processes or cell bodies. This result shows that treatment with angiogenic growth factors supports the maturation of blood vessels in cultured slices of the brain.

Real time analysis of neural stem cells in adherent cell culture shows that fate choice and other features of the differentiation in this cell lineage can be directly measured [44]. The data here extend the power of this approach to include the analysis of stem cells from different areas of the adult brain. The signaling pathways activated by angiogenic factors also allow the ex-vivo analysis of the interactions between blood vessels and neural cell types. This approach can be used to assess the properties of widespread multipotent precursors that are important in degenerative disease and cancer.

## Materials and Methods

### Cell Culture

E13.5 cortical embryonic mouse CNS stem cells were grown as previously described [31], [36]. Cells were expanded in serum-free DMEM/F12 medium with N2 supplement and FGF2 (20 ng/ml) for 5 days under 5% oxygen conditions and were re-plated fresh or from frozen stocks at 1,000–10,000 cells per cm^2^. FGF2 was added daily throughout the expansion phase, unless otherwise stated.

Adult rat (3–6 months old) SVZ Neural Stem Cell (NSC) cultures were grown in the same medium as the fetal cultures. SVZ and lateral forebrain tissue was collected from the areas shown in Fig. 2a, between approximately bregma +1.70 and −0.40 mm; midbrain tissue was collected between bregma −4.80 and −6.30 mm. The location corresponding to the sections used was determined by assessing the morphology of the SVZ, dentate gyrus, the third ventricle, and the third ventricle/aqueduct. Tissue was dissected from the areas described, triturated in 1 ml N2 medium containing FGF2 with a 1 ml pipette until no tissue clamps were seen; the triturate was allowed to settle for 1 min and the top 0.9 ml was collected, diluted in N2 containing FGF2 and plated. Under clonal density culture conditions, the maximal number of colonies in a 6-well plate was 12.

Treatments (Dll4 and Ang2, 500 ng/ml; FGF2, 20 ng/ml; JAK inhibitor, 200 nM) were every 2 days.

### Organotypic Slice Culture

For the preparation of the cortex-striatum-substantia nigra organotypic cultures, coronal sections (350–400 µm) from rat brains (Harlan Sprague Dawley, Indianapolis, IN) at postnatal day (PND) 0–2 were cut on a microslicer (D.S.K., Ted Pella, CA). Slices containing the striatum and the cortex were used for dissection of dorsolateral cortical and striatal tissue. For the substantia nigra (including pars compacta and pars reticulata), ventrolateral sections from mesencephalic slices were selected, and medial tissue regions were avoided. The tissue was arranged in serial order on a small rectangular piece of a Millicell-CM membrane (Millipore, Bedford, MA) with 20 µl of chicken plasma (Sigma, St. Louis, MO) on a coverslip. Then 20 µl of bovine thrombin (1000 National Institutes of Health units/0.75 ml; Sigma) was added. After plasma coagulation, the cultures were put into narrow culture tubes (Nunc, Naperville, IL), and medium was added (750 µl). The unbuffered standard medium consisted of 50% basal medium Eagle, 25% HBSS, and 25% horse serum with 0.5% glucose and 0.5 mM L-glutamine added (all Gibco, Grand Island, NY). After 3 and 27 d *in vitro* (DIV), 10 µl of mitosis inhibitor was added for 24 hr (4.4 mM cytosine-5-β-arabinofuranoside, 4.4 mM uridine, and 4.4 mM 5-fluorodeoxyuridine; calculated to final concentration; all Sigma). The medium was changed every 3–5 d.

### Magnetic Affinity Cell Sorting for AC133 (Prominin-1)

Dissociated E13.5 murine cortical tissue was resuspended in 300 ml of N2 containing medium without FGF2 or PDGF-AA. 30 µl of prominin-coated beads (Miltenyi Biotech, Auburn, CA) were mixed with approx. 6–10 million cortical cells and incubated for 40 minutes at 6°C. At the end of incubation, the cell suspension was placed on MS columns (Miltenyi Biotech) in the presence of a magnetic field. AC133- cells were eluted by washing twice with 1 ml N2 medium. The column was removed from the magnet and AC133+ cells were eluted by washing with N2 supplemented with bovine serum albumin (BSA) into a separate tube.

### Human Biopsies - Source

All human tissues in this study were obtained during surgical resections from patients with newly diagnosed or recurrent tumors. Materials in excess of pathological evaluation were used for research purposes in accordance with protocols approved by the Institutional Review Board of the National Institutes of Health. Written consent was obtained and all research tumor tissues were de-identified.

### Vertebrate Experiments (Rodents)

All research involving animals was conducted in accordance with NINDS ACUC (National Institute of Neurological Disorders and Stroke/Animal Care and Use Committee) guidelines and after their approval.

### Non-Human Primate Experiments

The study was conducted in accordance with the National Institutes of Health Guidelines on the use of animals in research and was approved by the Animal Care and Use Committee of the National Institutes of Neurological Disorder and Stroke [Animal Protocol ASP#1294-08; “Convection-enhanced delivery of stem cell inducing compounds into the primate brain”]. The animals were socially housed and provided with environmental enrichment and novel food items as provided for by the IACUC-approved NHP enrichment program. Potential postoperative pain was treated with ketoprofen IM, given preemptively at the time of surgery, and for 2 days post-operatively. Prophylactic antibiotic treatment and nursing care were also provided.

### Reagents

We used the following reagents and antibodies: FGF2 (233-FB), mouse Dll4 (1389-D4), CNTF (577-NT), Fibronectin (1030-FN), human angiopoietin-1 (923-AN), human angiopoietin-2 (623-AN), from R&D; JAK Inhibitor I (420099), from Calbiochem; Polyornithine (Sigma, P-3655), insulin (Sigma, I9278), Alexa-Fluor-conjugated secondary antibodies (Molecular Probes), DAPI (Sigma, D-8417), and general chemicals from Sigma.

For immunohistochemical staining, we used antibodies against the following markers: nestin (Chemicon, MAB353), Tuj1 (Covance, MMS-435P), GFAP (Dako, z0334 and Chemicon, MAB360), CNPase (Chemicon, MAB326), Sox2 (R&D, MAB2018); Hes3 (25393), Tie-2 (sc-324), Tie-2 (sc-31266), from Santa Cruz; α-tubulin (Sigma, T-6074); tyrosine hydroxylase (P80101 and P40101 from Pel-Freez); RECA-1 (MCA 970GA) from Serotec; pTie-2 (AF2720) from RnD Systems. Oregon Green – conjugated Phalloidin (07466) was purchased from Invitrogen.

### Statistical Analysis

Results shown are the mean ± s.d. Asterisks identify experimental groups that were significantly different (p-value, 0.05) from control groups by the Student's t-test (Microsoft Excel), where applicable. Statistical significance values are presented in Table S1.

## Supporting Information

Figure S1 Cultured NSCs express the angiopoietins. Fetal NSCs in culture under conditions that support self-renewal (FGF, FGF+Dll4 for 2 days) express Ang1 and Ang2. In contrast, conditions that rapidly induce their survival (FGF + CNTF, for 2 days) cause the loss of Ang2 (but not Ang1) expression.
(2.36 MB TIF)

Table S1 Statistical data set.
(0.03 MB DOC)
