# Supplementary figures and images for: Angiogenic Factors Stimulate Growth of Adult Neural Stem Cells
Source: PLoS One. 2010 Feb 26;5(2):e9414. doi: 10.1371/journal.pone.0009414 (PMC2829079; doi:10.1371/journal.pone.0009414)

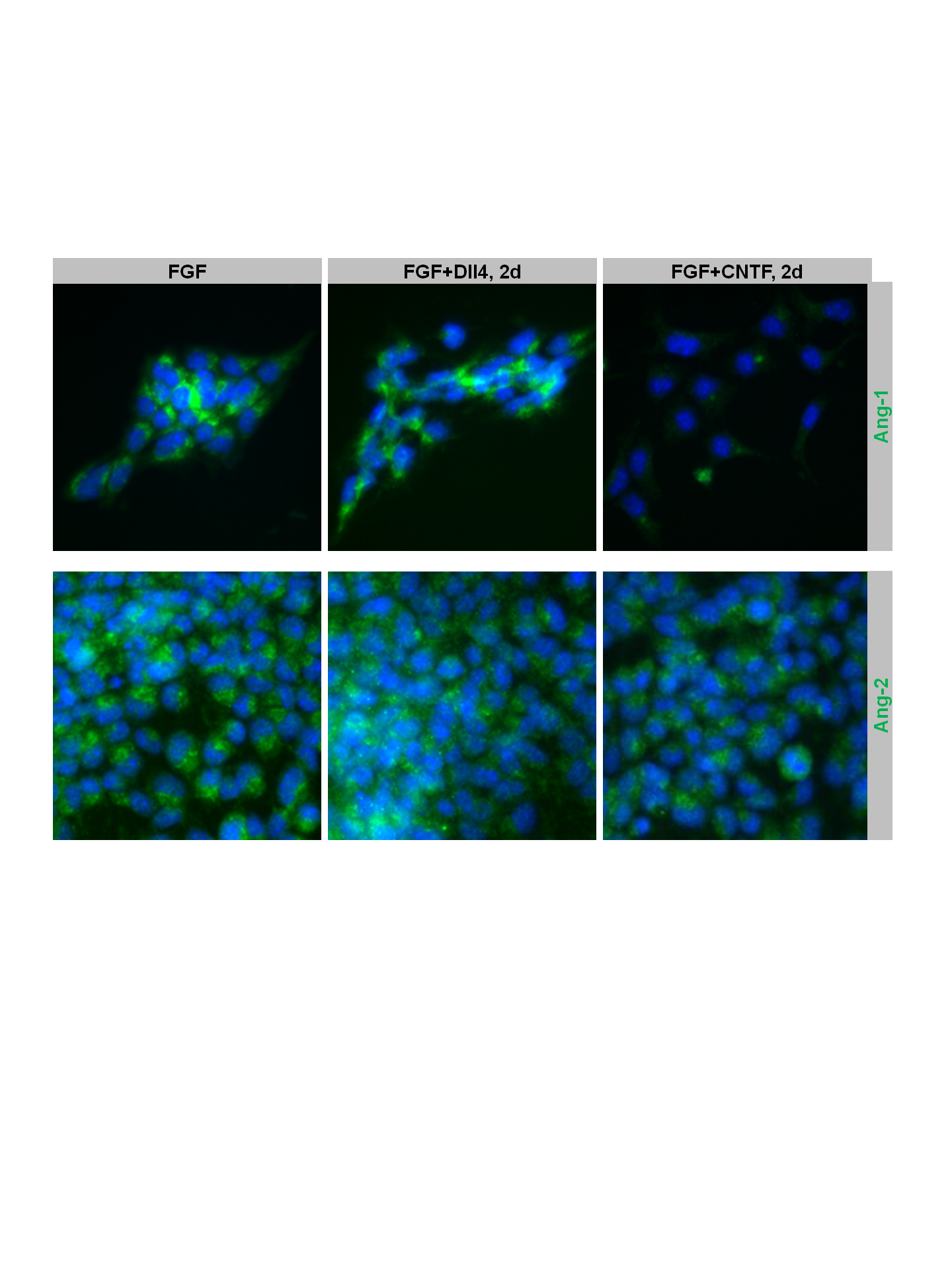

Supplement: Figure S1 — Cultured NSCs express the angiopoietins. Fetal NSCs in culture under conditions that support self-renewal (FGF, FGF+Dll4 for 2 days) express Ang1 and Ang2. In contrast, conditions that rapidly induce their survival (FGF + CNTF, for 2 days) cause the loss of Ang2 (but not Ang1) expression. (2.36 MB TIF) [file pone.0009414.s001.tif]
