# Supplementary material for: Angiogenic Factors Stimulate Growth of Adult Neural Stem Cells
Source: PLoS One. 2010 Feb 26;5(2):e9414. doi: 10.1371/journal.pone.0009414 (PMC2829079; doi:10.1371/journal.pone.0009414)
Supplement: Table S1 — Statistical data set. (0.03 MB DOC) [file pone.0009414.s002.doc]

**Table S1. Significance (p) values.**

| **Figure** | **p Value** | **Description** |
| --- | --- | --- |
| 1a | Dll4 from Dll4+Ang2: 2.15x10^-5^ | Cell#, rat SVZ |
| 1b | CT from Control: 0.036686 | Cell#, rat lateral forebrain |
| 1c | CT from Control: 0.005524 | Cell#, rat midbrain |
| 1d | CT from Control: 0.049 | Cell#, rat spinal cord |
| 1e | CT from Control: 0.015 | Cell#, monkey SVZ |
| 2b | CT from Control: 0.005154 | Striatal blood vessel area |
| 2c | CT from Control: 0.008552 | Striatal slice thickness |
| 2e | CT from Control: 0.018865 | Striatal slice TH signal |
